# Quantitative Investigation of the “K-Line Edge”, a Clustered K-Line (±) Group Derived from K-Line Assessment in Patients with Ossification of the Posterior Longitudinal Ligament

**DOI:** 10.3390/jcm14207339

**Published:** 2025-10-17

**Authors:** Kazuhiro Takeuchi, Kazunori Hosotani, Kensuke Shinohara, Kentaro Yamane, Shinichiro Takao, Shinnosuke Nakahara, Akiho Seki

**Affiliations:** 1 Department of Orthopedic Surgery, National Hospital Organization Okayama Medical Center, Okayama 701-1192, Japan; joker1011ks@yahoo.co.jp (K.S.); woodblocks0311@gmail.com (K.Y.); mechamecha_su_te_ki@yahoo.co.jp (S.T.); nakahara.shinnosuke.ep@mail.hosp.go.jp (S.N.); 2 Department of Integrated Science and Technology Mechanical Systems Course, National Institute of Technology, Tsuyama College, Okayama 708-0824, Japan; hosotani@tsuyama-ct.ac.jp; 3 Okayama Health Foundation, Okayama 700-0952, Japan; akiho.seki@gmail.com

**Keywords:** cervical spine, ossification of the posterior longitudinal ligament (OPLL), K-line, K-line edge, occupying ratio, cervical alignment, laminoplasty, posterior decompression, posterior correction, correction angle

## Abstract

**Background/Objectives:** We investigated the impact of ossification of the posterior longitudinal ligament (OPLL) and cervical alignment on K-line assessment, proposed the K-line edge category for transitioning from K-line (+) to (−), and demonstrated a quantitative evaluation using the K-line edge. **Methods:** The 268 patients with OPLL who underwent computed-tomography-based K-line assessment were retrospectively stratified into three groups [K-line (+)/(±)/(−)]. We graphically plotted their distributions based on the OPLL-occupying ratio (OPLL-OR) and cervical angle (θ C_2–7_). **Results:** The K-line (+), (±), and (−) groups comprised 159, 37, and 72 patients, respectively. The K-line (+) group demonstrated the lowest alignment value at −14.1°, suggesting a potential border for kyphosis at 14°. By examining the K-line (±) region, we successfully identified a clustered group and proposed the “K-line edge” for K-line (±), which was derived as y = 0.98x + 46.82 (R^2^ = 0.67). The K-line edge calculation depended on the OPLL-OR and θ C_2–7_ at each cervical level and determined the corresponding value for either OPLL-OR or θ C_2–7_. The slope of the K-line edge was almost horizontal at both ends and was steeper in the middle, with the alignment playing a dominant role in the mid-cervical region. The calculated borders were approximately at 12° kyphosis at C4-4/5 and at 11° at C5-5/6. **Conclusions:** Focusing on K-line (±) identified three major factors during the K-line assessment: the OPLL-OR, cervical alignment (θ C_2–7_), and cervical level. The K-line edge could be useful as a quantitative parameter for surgical decision-making.

## 1. Introduction

Ossification of the posterior longitudinal ligament (OPLL) can lead to neurological disorders. Two primary surgical approaches (anterior or posterior) have been debated. The anterior approach involves direct excision of the OPLL, resulting in direct decompression [1,2], which is essential in severe OPLL compression [3]. Moreover, this approach is effective for correcting kyphotic posture [4]. The anterior approach is expected to yield superior surgical outcomes; nonetheless, it is technically demanding [5,6]. The posterior approach involves decompression without fusion, commonly achieved through laminoplasty, which is a relatively safe and widely accepted surgery in Japan [5].

An indirect decompression mechanism is employed in the posterior approach, followed by posterior enlargement of the spinal canal [7]. Researchers have reported various risk factors for laminoplasty [8,9,10]. Both patient-related (background and clinical characteristics) and surgeon-related (years of experience and preference of surgical method) factors should be considered during surgical decision-making [11]. Among the various risk factors for laminoplasty, the magnitude of cord compression with OPLL and cervical alignment are considered two major risk factors [3,4,10,11].

Fujiyoshi et al. [12] introduced the K-line, a straight line connecting the midpoints of C2 and C7, as a method for simultaneously assessing OPLL size and cervical alignment. K-line (+) is defined as OPLL not intersecting the K-line in the ventral spinal cord, whereas K-line (−) indicates OPLL beyond the K-line. Researchers have reported unfavorable outcomes of posterior surgery in patients with K-line (−) [13]. Computed tomography (CT) remains indispensable for the diagnosis of OPLL, while magnetic resonance imaging is crucial for assessing the degree of spinal cord compression. While K-line measurement alone may not be adequate for comprehensive surgical decision-making, it remains widely accepted [14,15,16,17,18,19]. However, no study has examined the relationship between the K-line and OPLL compression and alignment.

**Figure 1:**
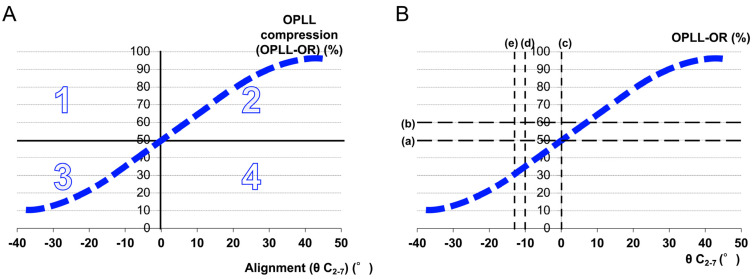
Relationship between cervical alignment (θ C2–7) and OPLL-occupying ratio (OPLL-OR), showing the distribution of K-line (+) and K-line (–) cases. (**A**) The blue line represents a conceptual distribution, generally aligning around OPLL-OR = 50% and cervical alignment = 0°. Since the spinal canal diameter and spinal cord thickness also influence this relationship, the actual distribution may shift slightly upward. Nevertheless, within the clinically common range (cervical alignment −20° to +20° and OPLL-OR values up to 60–70%), the distribution of K-line (±) cases tends to approximate a straight line. In particular, near the central inflection point (OPLL-OR = 50% and cervical alignment = 0°), the curvature of the distribution is small, and thus the line may appear nearly linear. The graph depicting OPLL compression (OPLL-OR) and cervical alignment (θ C_2–7_) is divided into four zones based on the combination of these values: (1: OPLL-OR >50%, θ C_2–7_ < 0°, 2: OPLL-OR > 50%, θ C_2–7_ > 0°, 3: OPLL-OR < 50%, θ C_2–7_ < 0°, 4: OPLL-OR < 50%, and θ C_2–7_ > 0°). (**B**) The threshold values (a, b, c, d, e) yield six possible combinations, but the plane is partitioned into four major regions. The graph depicting the θ C_2–7_ and OPLL-OR axes summarizes key numbers and is divided into four zones based on each combination of these threshold values: (a: OPLL-OR >50% [3], b: OPLL-OR > 60% [9,10,19,20], c: θ C_2–7_ < 0° [11,21], d: θ C_2–7_ < −10° [4], and e: θ C_2–7_ < −13° [22]). OPLL: ossification of the posterior longitudinal ligament; OPLL-OR: OPLL-occupying ratio (OPLL thickness divided by the spinal canal diameter); and θ C_2–7_: cervical alignment (angle between C2 and C7 according to the Cobb method).

**Figure 2:**
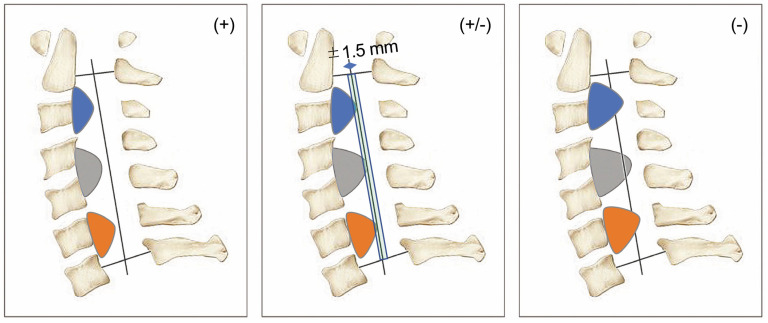
K-line classification. Evaluation through the K-line assessment. Patients with OPLL are divided into three groups as follows: (1) K-line (+); (2) K-line (±): K-line edge; and (3) K-line (−). The K-line (±) is defined within 1.5 mm of the top of the OPLL. OPLL: ossification of the posterior longitudinal ligament.

We aimed to demonstrate the impact of two major factors, OPLL compression and cervical alignment, on K-line assessment (Figure 1A); examine the inflection point [K-line (±), i.e., changes from K-line (+) to K-line (−)]; and explore key factors in K-line assessment (Figure 2).

## 2. Materials and Methods

This single-institution retrospective study enrolled 268 consecutive patients with OPLL who presented with cervical myelopathy, necessitating surgical intervention. The patients underwent preoperative radiography and computed tomography (CT; parameters: 120 kV, 100–200 mA, and 0.5 mm thickness for both slice data and reconstruction). This study was approved by the appropriate Institutional Review Board of National Hospital Organization Okayama Medical Center (RINKEN 2020-032). Informed consent was obtained in an opt-out fashion. This study was conducted between 6 August 2020, and 31 July 2022.

**Figure 3:**
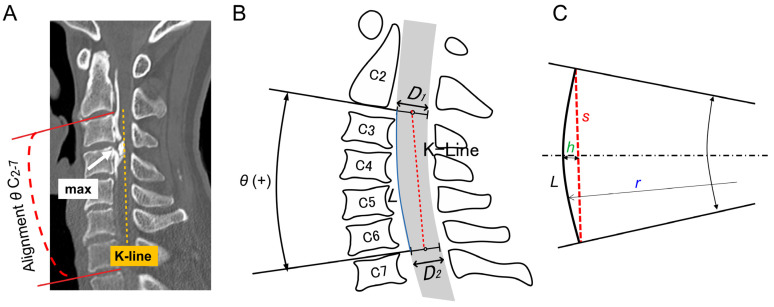
Image analysis. (**A**) Assessment of the OPLL volume, length, and thickness at the maximum OPLL level using computed tomography (CT). Measurement of the OPLL thickness and calculation of the OPLL-OR. Determination of the cervical alignment (θ C_2–7_). (**B**) Geometric model of the K-line (±). A geometric model for the K-line edge is formed with three major factors as follows: OPLL thickness (OPLL-OR), cervical alignment θ C_2–7_ and cervical level. Here, the cervical length is L, and the canal diameters at C2 and C7 are D1 and D2, respectively. (**C**) OPLL-OR calculation model/assuming that the cervical spine curve is an arc with arc length L, OPLL-OR can be calculated as OR = (0.5 + h/D) × 100 [%] from the ratio of the sagittal to the canal diameter D. CT: computed tomography; OPLL: ossification of the posterior longitudinal ligament; OPLL-OR: OPLL-occupying ratio (OPLL thickness divided by the spinal canal diameter); and θ C_2–7_: cervical alignment (angle between C2 and C7 according to the Cobb method).

Initially, cervical alignment (angle between C2 and C7 [θ C_2–7_]) was assessed using the Cobb method and maximum OPLL level. Lordosis and kyphosis are expressed as positive and negative values, respectively. We measured OPLL thickness at the maximum OPLL level and calculated the OPLL-occupying ratio (OPLL-OR), defined as OPLL thickness divided by the anteroposterior diameter of the spinal canal (Figure 3A). Subsequently, we evaluated OPLL using the K-line-based classification. In addition to the conventional K-line (+) and K-line (–) groups, we introduced the K-line (±) group (K-line edge), defined as cases in which the OPLL mass was located within 1.5 mm of the K-line. This category represents a borderline condition, where surgical decision-making is particularly challenging.

**Figure 4:**
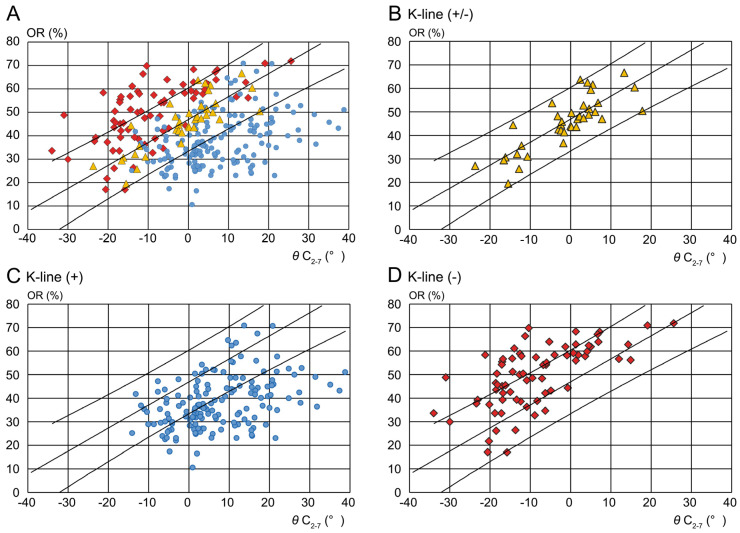
Distribution of patients with OPLL. (**A**) All cases plotted on a graph with the θ C_2–7_ and OPLL-OR axes. The distribution of the 95% confidence ellipses indicates that the segmental position of each K-line group shifts to the lower right in the following order: (−), (±), and (+). OPLL: ossification of the posterior longitudinal ligament; OPLL-OR: OPLL-occupying ratio (OPLL thickness divided by the spinal canal diameter); and θ C_2–7_: cervical alignment (angle between C2 and C7 according to the Cobb method). (**B**) K-line (±) distribution/distribution of the K-line (±) indicates the inflection point between the K-lines (+) and (−). The linear correlation line is termed the K-line edge line. The K-line edge is obtained as y = 0.98x + 46.82, and R^2^ = 0.67. (**C**) K-line (+) distribution with the K-line edge line/K-line (+) has the lowest value of cervical alignment at −14.1°. Therefore, 14° kyphosis may be the border between the K-lines (+) and (−) in the total assessment. (**D**) K-line (−) distribution with K-line edge line/kyphosis is a dominant factor in several K-line (−) cases. Yellow triangle: K-line (±), Blue circle: K-line (+), Red diamond: K-line (−).

Three senior spine surgeons independently evaluated the K-line classification. After classifying evident K-line (+) and (−) cases, a ±1.5-mm width from the K-line was assigned as the K-line edge (±). We reviewed identical images from 50 patients to ensure interobserver agreement, and the Fleiss kappa coefficient of the evaluations by the three physicians was 0.73. Despite some variation in K-line (±) assessment, the data were considered reliable. Based on K-line assessment, the participants were classified into the K-line (+), K-line edge (±), and K-line (−) groups (Figure 2). We presented the distribution of these K-line groups graphically based on the OPLL-OR and θ C_2–7_ axis (Figure 4A). A linear approximation line and 95% confidence ellipse were added to the K-line (+, ±, −) scatter diagram. The plot of confidence ellipses, Pearson correlation coefficients, and slope and intercept of the linear approximation line were calculated using Origin Pro (OriginLab Corporation, Northampton, MA, USA). We investigated the characteristics of each K-line group at the cervical level.

In this study, we focused on the K-line edge (±), the transitional region between K-line (+) to K-line (−), as well as identifying a relatively consolidated, unique population forming a narrow band (Figure 4B). Factors involved in the K-line edge evaluation were also investigated.

## 3. Results

### 3.1. Participant Demographics

Among 268 participants (198 men and 70 women; mean age at surgery: 66.3 (35–86) years) with OPLL, the mean follow-up duration was 5.1 years; Table 1 presents the demographic data. The maximum OPLL levels were C2-2/3 (N = 25), C3-3/4 (N = 44), C4-4/5 (N = 52), C5-5/6 (N = 105), C6-6/7 (N = 34), and C7-C7/T1 (N = 8). The alignment (θ C_2–7_) ranged from −34.0° (kyphosis) to 38.8° (lordosis). The mean OPLL-OR was 42.2% (10.6–71.9%).

### 3.2. Distribution of Each K-Line Group

Using the θ C_2–7_ and OPLL-OR axis, we plotted the distribution of each OPLL case (Figure 4A). The K-line (+), (±), and (−) groups comprised 159, 37, and 72 patients (Table 1), with Pearson coefficients of 0.400, 0.818, and 0.639, respectively. The K-line (±) plot displayed a narrow band.

In the K (±) group, a close positive correlation was found between the C2-C7 angle and compression rate. The regression line and 95% prediction interval (PI) are illustrated in Figure 4B. In this series, the PIs obtained from statistical analysis encompass all but one K-line (±) case, indicating that this K-line (±) group was relatively cohesive and unique. Therefore, we focused on the K-line (±) distribution to elucidate the changing point between K-lines (+) and (−). Examination of the area outside this 95% PI region showed that cases below the lower 95% PI limit were entirely K-line (+) (lower right; Figure 4A, C), whereas those above the upper 95% PI limit were solely K-line (−) (upper left; Figure 4A,D).

Furthermore, the K-line (+) group had the lowest value of cervical alignment at −14.1° (Figure 4C), whereby 14° kyphosis may be the critical value between K-lines (+) and (−) for overall assessment.

### 3.3. K-Line Edge Line at Each Cervical Level

**Figure 5:**
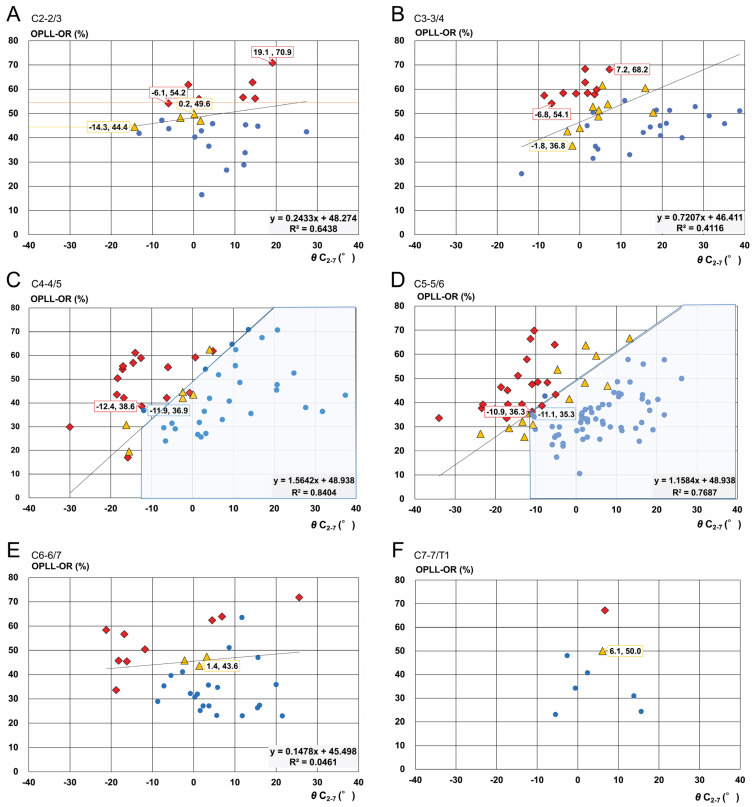
K-line edge line at each cervical level. Distribution of each K-line group. (**A**) C2-2/3 /slope of the K-line edge is almost horizontal. The OPLL-OR is the dominant factor. (**B**) C3-3/4/K-line (+), (±), and (−) groups are precisely aligned. The slope of the K-line edge is 0.72. (**C**) C4-4/5/inclination of the K-line edge line is 1.56. The maximum kyphotic angle in the K-line (+) group is 11.9°. Furthermore, all the cases with angles < −12.4° belong to the K-line (−) group. (**D**) C5-5/6/slope of the K-line edge line is 1.16, between C3-3/4 and C4-4/5. K-lines (+) and (−) are close to the correlation line. The inflection point is defined as the meeting point at which these two intersect the K-line edge. The changing point between K-lines (+) and (−) is 11.0° kyphosis. (**E**) C6-6/7 /slope of the correlation line is almost horizontal, similar to that of C2-2/3. (**F**) C7-C7/T /a small number and large deviation are observed. Yellow triangle: K-line (±), Blue circle: K-line (+), Red diamond: K-line (−).

The distribution of each K-line group is shown in Figure 5 and the K-line edge line in Table 2. The upper margin was C2. Despite the limited data, the K-line edge at C2-2/3 was y = 0.24x + 48.27. The slope of the K-line edge at C2-2/3 was almost horizontal, which was not affected by the alignment, and OPLL-OR was dominant in the K-line assessment. The minimum K-line (−) value was −54.2%. The K-line (±) group had an OPLL-OR >44.4% (Figure 5A). The C3-3/4 distribution exhibited a sharp slope, and the three groups were precisely lined. The slope of the K-line edge was 0.72. The remaining two groups, K-lines (+) and (−), were arranged obliquely along this slope (Figure 5B). The plots at C4-4/5 demonstrated a similar distribution; however, the inclination of the K-line edge line was 1.56°, which was almost two-fold higher than that of C3-3/4. The maximum kyphotic angle in the K-line (+) group was 11.9°. Furthermore, all patients with less than −12.4° belonged to the K-line (−) group. In this series, the changing point was approximately −12° between K-lines (+) and (−) (Figure 5C). The C5-5/6 group included 104 patients (the largest group), and this group exhibited a similar shape to that of the C4/5 group. The slope was 1.16 between C3-3/4 and C4-4/5. In this series, K-lines (+) and (−) were close to the following correlation lines: K-line (+): −11.1°, 35.3%; K-line (−): −10.9°, 36.3%; and K-line edge line: y = 1.16x + 48.89. The inflection point was defined as the meeting point at which K-lines (+) and (−) intersected the K-line edge. At this level, 11.0° kyphosis represented the changing point (Figure 5D). For C6-6/7, the slope of the correlation line was almost horizontal, similar to that of C2-2/3. C6-6/7 and C7-T1 evaluation yielded small sample sizes and large deviations (Figure 5E,F).

### 3.4. Slope of the K-Line Edge Line at Each Cervical Level

**Figure 6:**
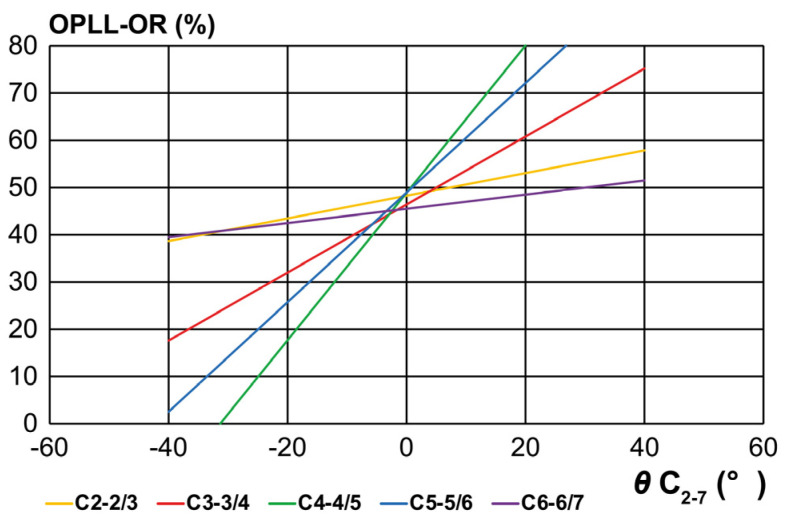
Slope of the K-line edge line at each cervical level. The K-line edge slope is gentle at both ends and steep in the middle. The slope at C2-2/3 gradually increases to C3-3/4 and C4-4/5 and decreases to the lower end at C6-6/7.

The regression line for each cervical level is shown in Figure 6 and Table 2, with the differences in the slopes possibly reflecting the differences in the K-line (±) boundary values depending on the cervical level. The slope was mild at both ends and steep in the middle. The slope at C2-2/3 gradually increased to C3-3/4 and C4-4/5 and decreased to the lower end of C6-6/7. The mid-cervical level displayed steep inclination, and alignment was the dominant factor affecting K-line assessment.

## 4. Discussion

The unique contribution of this study is the quantitative evaluation of the K-line edge, which expands the conventional binary classification of the K-line. This approach provides new insights into the prognostic utility of the K-line in surgical decision-making.

Posterior decompression and laminoplasty are the most common procedures for cervical OPLL treatment in Japan, despite the associated postoperative complications, such as axial pain and C5 nerve palsy [23]. Hirabayashi et al. reported that clinicians prefer open-door laminoplasty [24]. Several risk factors have been reported after the posterior procedure [3,4,8,9,10,11], including dynamic factors (e.g., segmental instability) [25,26]. Moreover, OPLL exhibits a progressive profile that may affect the surgical outcomes during follow-up [27,28].

OPLL size and cervical alignment are the two major risk factors. Large OPLL is associated with poor clinical outcomes [21,29], with 50% [3] and 60% OPLL-OR [9,10,19,20] constituting an index of poor prognosis. Consequently, the anterior procedure is performed in 50–60% of OPLL-OR cases (Figure 1B).

Batzdorf and Batzdorff [30] analyzed spinal curvature and evaluated the relationship between alignment and outcome after cervical laminectomy. In this study, the distribution of K-line (+/–) cases was demonstrated on the plane defined by θ C_2–7_ and OPLL-OR (Figure 1). Although the plotted line is conceptual rather than a strict regression, it serves as a clinical guide. Importantly, near the central inflection point (OPLL-OR = 50% and cervical alignment = 0°), the curvature is small, and the distribution appears nearly linear. This supports the practical utility of approximating the distribution as a straight line within the clinically common range (−20° ≤ θ C_2–7_ ≤ +20°, OPLL-OR ≤ 60–70%). Indirect decompression through laminoplasty is easily affected by residual OPLL, particularly in the kyphotic posture. Preoperative kyphosis (0°, 10°, and 13°) has been reported as a risk factor (Figure 1B) [4,11,21,22]. These threshold reports are summarized using the alignment (θ C_2–7_) and compression (OPLL-OR) axes in a graph. Moreover, while two thresholds for OPLL-OR and three for alignment yield six possible combinations, the graphical partition is determined by one vertical and one horizontal threshold line, resulting in four major regions (Figure 1B).

Nonetheless, the K-line track in the OPLL-OR-alignment relationship was unelucidated. Our study provides important insight into the relationship between K-line evaluation, OPLL-OR, and cervical alignment, which graphically represents each patient’s condition (Figure 1A). We inductively pursued the factors of K-line evaluation. In the K-line (+) group, the lowest cervical alignment was −14.1°. This value may be crucial for reversal from a positive to a negative state, crossing the K-line (Figure 4C). Our results are consistent with those of Suda et al. [22], who identified 13° of kyphosis as a crucial cutoff point.

Originally, K-line assessment was a qualitative analysis (+ or −) and comprised no quantitative components. The focus on this inflection point led to a more definite picture of the K-line, graphically depicted with the OPLL-OR and θ C_2–7_ axis. The study population exhibited a heterogeneous distribution, with a high proportion of K-line (+) cases, a low proportion of (−) cases, and an even lower proportion of (±) cases. This is a common feature expected across institutions. Despite heterogeneous patient distribution, our findings highlight the clinical importance of quantitative K-line edge assessment for OPLL management. The K-line (±) group distribution formed a relatively narrow band that did not appear to be affected by the sampling method or sample size. Thus, this unique population formed a cluster (R^2^ = 0.67) that was dependent on the OPLL-OR and θ C_2–7_. We proposed a K-line edge category for the K-line (±) group, which determines the dividing edge between the K-line (+) and (−) groups, and calculated the linear correlation of K-line (±) (K-line edge line: y = 0.98x + 46.82) (Figure 4B).

The linearity of the scatterplot can be confirmed by the relational expression between the cervical curve and the compression rate. Harrison et al. [31] proposed a cervical sagittal plane model using arcs and reported its predictions and validity based on actual measurements.

In the arc model of the sagittal cervical spine, shown in Figure 3B,C, the sagittal height, *h*, affecting the OPLL-OR in the K-line, is expressed as

$$h=r-\sqrt{r^{2}-{\left(\frac{s}{2}\right)}^{2}}$$

where *r* is the radius, *s* is the chord length, and *h* is the sagittal angle. Because the radius, *r*, varies with *θ*, the above equation can be rewritten using the known arc length, *L*, as

$$h=\frac{L}{\theta }\left\{1-\sqrt{{\left(cos\frac{\theta }{2}\right)}^{2}}\right\}$$

In this equation, the variation in the cosine component is small, within the range of ±40° of θ, which makes the proportional component effect more significant. Thus, the OPLL-OR exhibits a near-linear distribution with respect to changes in θ C_2–7_ (Figure 1A). Our results showed that the K-line edge (±) group formed a straight line, which was consistent with the results obtained from the model. Furthermore, the 95% PI involved all but one K-line edge case (Figure 4B), which indicates a relatively cohesive and unique group. Therefore, we focused on the K-line (±) distribution to elucidate the transitional point between K-lines (+) and (−).

Subsequently, we assessed changes from K-line (+) to (−) using the K-line edge line at each cervical level (Table 2 and Figure 5). These subgroup analyses provided a more definite picture of the K-line edge trajectory (Figure 5). K-line (−) is expected to begin at 50% OPLL-OR, irrespective of cervical alignment. The K-line edge was almost horizontal at the upper end (C2-2/3). This distribution was unaffected by cervical alignment, and OPLL-OR was the only factor in the case of a straight cervical spine. The minimum values for K-lines (−) and (±) were 54.2% and 44.4%, respectively (Figure 5A). Our data support the critical values of OPLL-OR reported previously [3,4,9,10,11,19,20,21,22].

Particularly in mid-cervical lesions, we observed denser populations (R^2^: 0.84 for C4-4/5 and 0.77 for C5-5/6). The assessment at each level yielded 12.1° and 11.0° kyphosis at C4-4/5 and C5-5/6, respectively, as the critical values (Figure 5C,D). These data were close to those of our total analysis (14.1° kyphosis) and a previous report (13° kyphosis) [22]; nonetheless, our values were marginally lower. Thus, cervical alignment may be a dominant factor at the mid-cervical level. Taken together, the influence of the cervical level, i.e., the region of interest, was manifested in the K-line assessment.

From a clinical perspective, the K-line (±) group represents a borderline category where surgical planning is particularly challenging. It may also provide a practical reference for posterior corrective fusion surgery, where achieving alignment within this range could serve as a safer target and help avoid neurological complications, in line with the findings reported by Koda et al. K-line edge assessment offers a valuable tool for informed surgical planning by providing a quantitative measure of the inflection point between K-lines (+) and (−). The anterior approach remains a rational treatment option for K-line (−) cases, particularly those with massive OPLL. Nevertheless, cervical screw fixation offers another solution. Compared with the anterior approach, the posterior approach offers equivalent neurological recovery without serious complications [14,32]. Posterior fusion with instrumentation, initially used to stabilize and retain the cervical alignment [33,34], may effectively address dynamic factors contributing to poor surgical outcomes [8,25,26]. Moreover, postoperative loss of lordosis or kyphotic changes may affect the clinical course [8,10,11,35]. Posterior instrumentation surgery is used to prevent postoperative kyphosis in the straight spine [36]. Finally, we transition toward a more aggressive correction method with instrumentation, based on backshift and stabilization of the spinal cord [7]. Koda et al. [37] reported that correction from negative to positive was preferable for a better surgical outcome in the K-line (−) group. This finding emphasizes the significance of the K-line edge concept as a transition point between K-lines (+) and (−), consistent with the findings of Koda [37].

The optimal correction for kyphosis remains to be elucidated [37]. Several surgeons have expressed concerns regarding excessive correction with the cervical screw fixation approach because this aggressive approach is associated with a risk of iatrogenic nerve kinking or irritation, followed by segmental palsy [38,39]. Hojo et al. [38] reported that 17.6° per fused segment is the critical value for correction. Therefore, we recommend applying a mild correction force up to a straight or marginally lordotic curvature.

Our K-line edge assessment can facilitate the quantitative evaluation of posterior corrective surgery. We calculated the target angle using the K-line edge formula y = 0.98x + 46.82 (Figure 4B, Table 2). Furthermore, the K-line edge formed a fine band depending on the cervical level. Thus, our K-line edge evaluation had three elements: OPLL-OR, θ C_2–7_, and cervical level. Using this K-line edge formula, we calculated the specific values of OPLL-OR or θ C_2–7_ at each cervical level using these three elements (Table 2). For example, 56.6% OPLL-OR was expressed as the inflection point at 10° of lordosis (θ C_2–7_ = 10°); 46.8% in a straight spine and 37.0% with 10° kyphosis. Further, each cervical level had a unique K-line edge distribution and fixed critical number of alignments and OPLL-OR. The critical OPLL-OR values at 10° of lordosis were 64.5% and 60.5% for C4-4/5 and C5-5/6, respectively (Table 3).

Figure 6 shows the slopes of the K-line (±) regression lines at each level. These differences in slope may reflect the following clinical perspectives: (1) the K-line edge depends on the cervical level; (2) the slope of the K-line edge was mild at both ends and steep in the middle, indicating alignment as the dominant factor in the mid-cervical spine; and (3) it may indicate corrective targets (from K-line (−) to K-line (+)) at each cervical level. A larger number of cases is required for further study.

We focused on elucidating the boundary value of the K-line (±) and characterizing the unique features. This novel report demonstrated the inflection point between K-lines (+) and (−). K-line (±) is a unique group, and we propose that it should be designated as K-line edge. The K-line edge assessment covered the thresholds reported to date.

Although the K-line is a well-recognized prognostic factor in posterior interventions [12,13,14,15,16,17,18,19], the K-line approach still has its exceptions and limitations [40]. These exceptions may arise from the limitation of relying solely on qualitative assessment with (+) and (−) or not considering the cervical level.

Notably, this K-line edge assessment provided the quantitative aspect of the previous qualitative evaluation. The K-line edge indicates the point of change from (+) to (−). Thus, the K-line edge assessment had the following three elements: OPLL-OR, θ C_2–7_, and cervical level.

Moreover, OPLL exhibits a progressive profile that may affect surgical outcomes during follow-up [11,27,28]. Despite the inability to control the OPLL course, we could control the alignment during surgery. The K-line edge suggests an ideal correction value (θ C_2–7_) for successful surgical treatment at each OPLL-OR and cervical level. Furthermore, this assay can measure OPLL-OR and alignment bidirectionally, suggesting an ideal corrective value based on alignment or compression rate for each fusion case. The K-line edge assessment is expected to be useful as a grading scale in OPLL surgery.

This study has several limitations. First, the retrospective design may have introduced selection bias. Second, the sample size was modest, and the distribution between groups was uneven. Third, clinical outcomes were not validated in this study. Future prospective studies with larger cohorts and long-term follow-up are warranted to confirm the present findings.

## 5. Conclusions

We summarize the findings of the K-line edge evaluation as follows:

(1) K-line (+) is not observed in cases with a kyphosis of −14° or less (cases with a kyphosis of 14° or more). This value is similar to previously reported value (13° kyphosis).
(2) The K-line evaluation has three essential elements: OPLL thickness, cervical alignment, and cervical level at which the evaluation is performed, extending beyond the conventional binary classification of K-line (+) and (–).
(3) The K-line should be considered a prognostic rather than a diagnostic tool. The K-line edge assessment introduces a quantitative aspect, which is expected to be useful as a grading scale for closely focusing on the correction angle targeting at least K-line edge (K-line (±), thereby helping to avoid excessive correction and the risk of neurological complications.

## Figures and Tables

**Table 1:** Demographic Data.

Level	Cases	K-Line			Level	Alignment (θ C_2–7_)		OPLL-OR	
		(+)	(±)	(−)		Average	Range	Average	Range
C2–2/3	25	14	4	7	C2–2/3	4.5	−14.3–27.4	45.8	16.5–70.9
C3–3/4	44	20	10	14	C3–3/4	6.6	−31.0–38.8	48.8	25.2–68.3
C4–4/5	52	30	6	16	C4–4/5	0.9	−31.0–38.8	44.4	17.0–70.9
C5–5/6	105	67	13	25	C5–5/6	−0.7	−34.0–26.2	38.1	10.6–69.8
C6–6/7	34	22	3	9	C6–6/7	1.8	−21.2–25.6	40.6	23.0–71.9
C7–7/T1	8	6	1	1	C7–7/T1	4.5	−5.5–15.6	39.9	23.1–67.2
Total	268	159	37	72	Average	1.8°	—	42.2%	—

We retrospectively reviewed 268 patients with OPLL (198 men and 70 women). (1) K-line (+): 159 patients; (2) K-line (±) (K-line edge): 37 patients; and (3) K-line (−): 72 patients. OPLL: ossification of the posterior longitudinal ligament, OPLL-OR: occupying ratio, and alignment (θ C_2–7_)_:_ angle between C2 and C7.

**Table 2:** K-line edge line: a correlation linear line of K-line (±) at each cervical level.

Level	K-line (+/−): K-Line Edge Line	Coefficient	R^2^
Total	y = 0.98x + 46.82	0.98	0.67
C2-2/3	y = 0.24x + 48.27	0.24	0.64
C3-3/4	y = 0.72x + 46.41	0.72	0.41
C4-4/5	y = 1.56x + 48.89	1.56	0.84
C5-5/6	y = 1.16x + 48.94	1.16	0.77
C6-6/7	y = 0.15x + 45.50	0.15	0.05
C7-C7/T1	—	—	—

The K-line edge at C2-2/3 was y = 0.24x + 48.27, which was almost horizontal. The C3-3/4 distribution exhibited a sharp slope. The plots at C4-4/5 demonstrated a similar distribution; however, the inclination of the K-line edge line was almost two-fold higher than that of C3-3/4. The C5-5/6 group exhibited a shape similar to that of the C4/5 group. The slope was 1.16 between C3-3/4 and C4-4/5. For C6-6/7, the slope was almost horizontal, similar to that of C2-2/3.

**Table 3:** Critical OPLL-OR boundaries depending on the cervical alignment and cervical level.

Level	K-Line Edge Line	Alignment (θ C_2–7_)				
		Kyphosis −10°	Straight 0°	Lordosis		
				10°	13.5°	20°
Total	y = 0.98x + 46.82	37.0	46.8	56.6	60.0	66.4
C4-4/5	y = 1.56x + 48.89	33.3	48.9	64.5	—	80.1
C5-5/6	y = 1.16x + 48.94	37.3	49.0	60.5	—	72.1

The K-line edge, which is the linear correlation line of the K-line (+/−), was calculated at each cervical level. This formula suggests an approximate critical boundary of the OPLL-OR depending on the cervical alignment. OPLL-OR: occupying ratio; alignment (θ C_2–7_)_:_ angle between C2 and C7.

## Data Availability

The datasets generated and analyzed during the current study are not publicly available due to privacy concerns and ethical restrictions but are available from the corresponding author upon reasonable request.

## Abbreviations

The following abbreviations are used in this manuscript:

CT	computed tomography
OPLL	ossification of the posterior longitudinal ligament
OPLL-OR	OPLL-occupying ratio
